# New Numbering System for Teeth Following Hemisection, Bicuspidisation, and Root Resection

**DOI:** 10.1016/j.identj.2024.12.028

**Published:** 2025-01-24

**Authors:** Manali Ramakrishnan Srinivasan, Saravanan Poorni, Liran Levin, Paul MH Dummer, Venkateshbabu Nagendrababu

**Affiliations:** Department of Conservative Dentistry and Endodontics, Sri Venkateswara Dental College and Hospital, Chennai, India; College of Dentistry, University of Saskatchewan, Saskatoon, Saskatchewan, Canada; School of Dentistry, College of Biomedical and Life Sciences, Cardiff University, Cardiff, UK; Department of Restorative Dentistry, College of Dental Medicine, University of Sharjah, Sharjah, United Arab Emirates

Dear Editor

Tooth numbering systems are essential for the accurate identification of teeth in clinical practice, research, and education. Accurate notation is not merely a matter of convenience but also directly influences communication, patient care, treatment outcomes, and the integrity of dental research. The existing tooth numbering systems, such as the Universal Numbering System and the Fédération Dentaire Internationale (FDI) system, have been used for many years. The Universal Tooth Numbering System (also known as the Universal Dental Notation or U.S. System) is primarily used in the United States to identify and label teeth.^1^ The Fédération Dentaire Internationale (FDI) World Dental Numbering System is a widely accepted and well-established 2-digit notation.^2^

Although these systems have been in use for many years, Rajendra Santosh and Jones^3^ suggested the inclusion of a ‘dot’ between the two numerals of the FDI system to differentiate the sextant number from the tooth number. For example, the maxillary left second molar historically documented as tooth 27 in the FDI notation is always pronounced as ‘two-seven’ and not ‘twenty-seven’, so as to prevent potential confusion with the Universal System where tooth 27 is the mandibular right canine. Rajendra Santosh and Jones^3^ suggested that tooth 27 in the FDI system should be written as 2.7 with similar modifications to all teeth using that system.

Although the existing tooth numbering systems are the most accepted format of tooth notation, they cannot be applied to clinical situations such as hemisection, bicuspidisation, and root resection, which are performed on multi-rooted teeth to separate them into various fragments/roots.^4^ This poses a unique challenge for the accurate identification of the remaining tooth roots post-procedure for documentation, treatment planning, follow-up, and communication with other professionals. The proposed numbering system is a modification of the current numbering systems that will resolve this limitation while maintaining accuracy and simplicity.

## Challenges with the current numbering systems

The current tooth numbering systems are not able to take account of hemisection, bicuspidisation, or root resection as they are not able to define the remaining tooth/root(s). Hemisection is undertaken to remove the crown and root of one-half of a mandibular molar while the remaining tooth crown and root is restored into a premolar shape. Bicuspidisation is undertaken to divide a mandibular molar into two halves with each section then being restored as individual teeth (roots). Root resection is undertaken to remove an entire root that has periodontal or endodontic complications that are not amenable to more conservative approaches.

Currently, no tooth numbering system can indicate or define the remaining tooth/root sections following these procedures, making it necessary to rely on a full written explanation. As a consequence, it is impossible for clinicians to define the remaining tooth sections when using the current numbering systems. This has the potential to cause errors in communication among dental practitioners for accurate record-keeping and follow-up.

## Need for clarity in clinical documentation

Clear identification of the remaining portions of the teeth following hemisection, bicuspidisation, and root resection is essential for treatment planning, particularly when decisions regarding restoration or extraction are made in future. Accuracy and clarity in documentation will aid in follow-up care and ensure that all clinicians who are involved are aware of the specifics of the case and the status of the relevant tooth fragments.

## Proposed new numbering system for teeth following hemisection, bicuspidisation, and root resection by modifying the current numbering systems

**Fig. 1:**
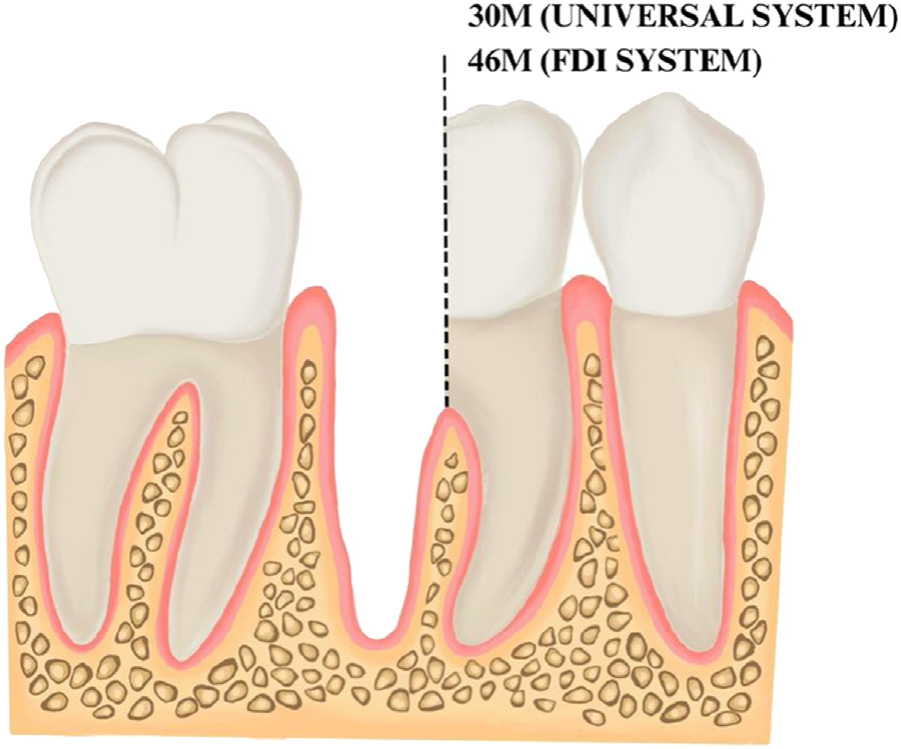
Illustration of the new numbering code for hemisection. The retained mesial root of a mandibular right first molar is indicated as 30M in the modification to the Universal System and indicated as 46M (or 4.6M) in the modification to the FDI system. The Bold text is used to highlight the new numbering system that is proposed.

**Fig. 2:**
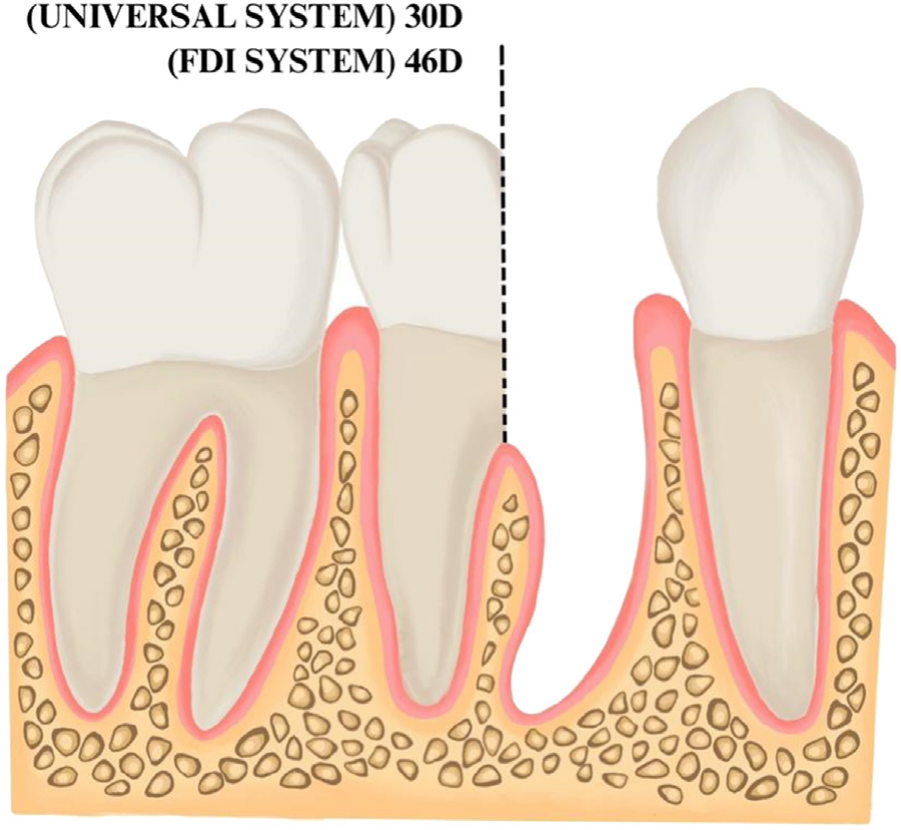
Illustration of the new numbering code for hemisection. The retained distal root of a mandibular right first molar is indicated as 30D in the modification to the Universal System and indicated as 46D (or 4.6D) in the modification to the FDI system. The Bold text is used to highlight the new numbering system that is proposed.

**Fig. 3:**
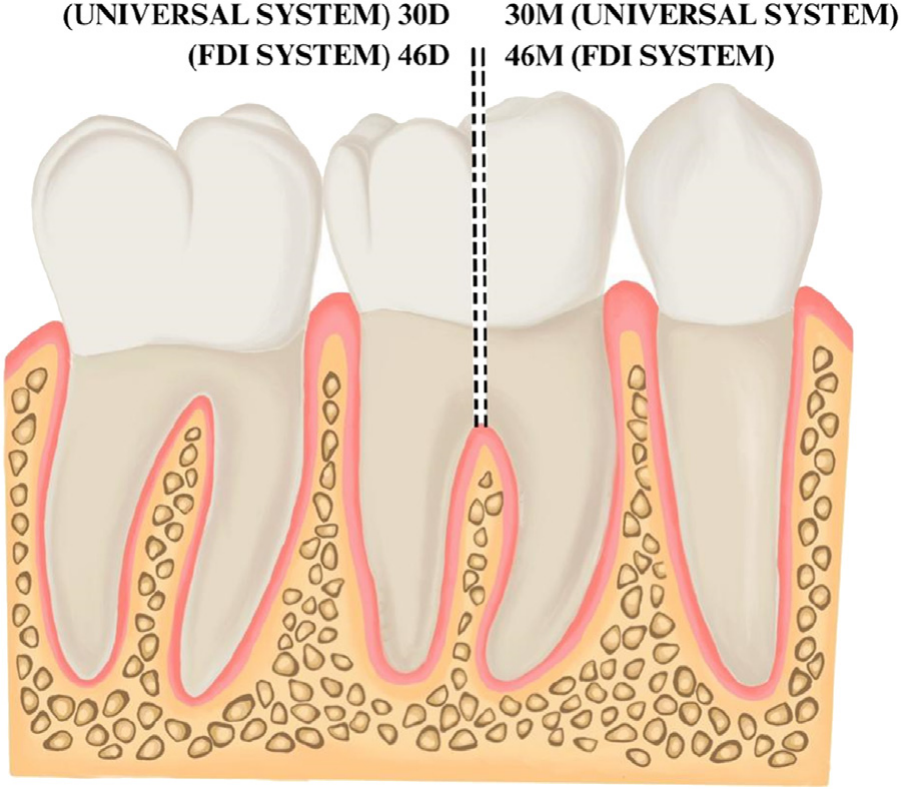
Illustration of the new numbering code for bicuspidisation. The retained mesial and distal roots of a mandibular right first molar are indicated as 30D and 30M in the modification to the Universal System and indicated as 46D and 46M (or 4.6D and 4.6M) in the modification to the FDI system. The Bold text is used to highlight the new numbering system that is proposed.

**Fig. 4:**
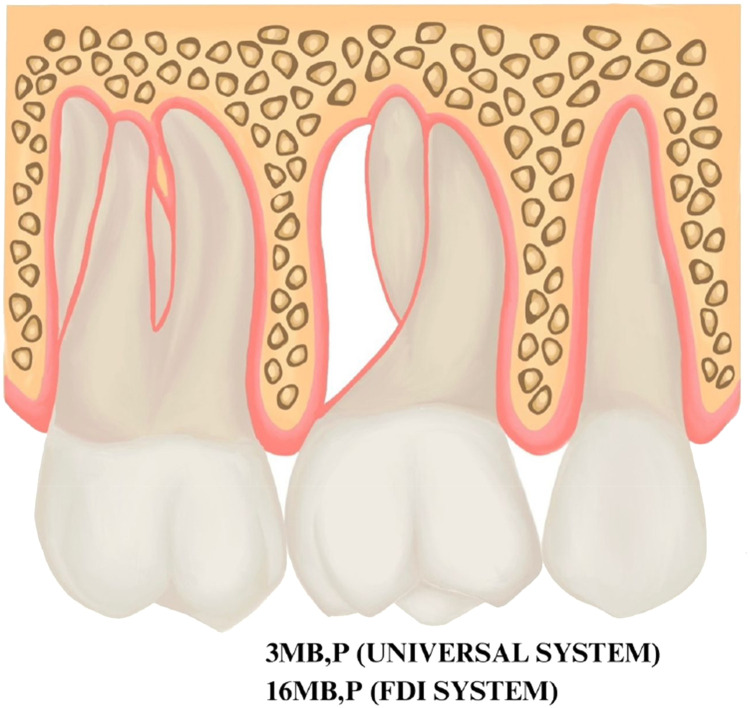
Illustration of the new numbering code for root resection. The retained mesial-buccal and palatal roots of a maxillary right first molar are indicated as 3MB,P in the modification to the Universal System and indicated as 16MB,P (or 1.6MB,P) in the modification to the FDI system. The Bold text is used to highlight the new numbering system that is proposed.

The proposed new numbering system for teeth following hemisection, bicuspidisation, and root resection is a modification of the existing numbering systems using a simple yet effective code to indicate the presence of each remaining tooth/root section following hemisection (Figures 1 and 2), bicuspidisation (Figure 3), and root resection (Figure 4). The code is designed to be intuitive and easy to incorporate into current practice and electronic health records. The base numbering system for bicuspidisation, hemisection, and root resection cases will follow the Universal and FDI systems with a minor modification to represent the remaining tooth sections.

To specifically indicate hemisection, bicuspidisation, and root resection a code is added to the standard tooth number in both the Universal and FDI systems that describes the remaining root(s) of the tooth (Table 1). This is essential because these procedures often involve the removal of one root, leaving the other *in situ,* or involve retaining all roots as individual tooth/root fragments.

**Table 1:** New tooth numbering codes for mandibular and maxillary molars.

Mandibular molars	Maxillary molars
**Code M indicating Mesial root**: When the mesial root of the tooth is retained the tooth number will be followed by ‘M’. **Code D indicating Distal root**: When the distal root of the tooth is retained the tooth number will be followed by ‘D’.	**Code P, MB, and DB indicating palatal, mesio-buccal, and disto-buccal roots**: When one or more of the roots of a maxillary molar are retained, the tooth number will be followed by the relevant root code(s), ‘P’, ‘MB’, and ‘DB’.

Although hemisection and bicuspidisation are most frequently performed on mandibular molars, these procedures and root resection can be performed on maxillary molars as well. For example, there are clinical reports that describe the management of four rooted maxillary molars where hemisection was the treatment of choice.^5^ In such clinical situations, the new numbering system can be applied to denote the retained segment(s) of the tooth.

## Conclusions

The suggested modification to the existing tooth numbering systems provides a clear method for identifying and documenting teeth following hemisection, bicuspidisation, and root resection. The new system will improve communication, patient understanding, and help in effective treatment planning and follow-up. By introducing the modification, clinicians can better communicate cases involving partial tooth retention, leading to better outcomes and more efficient care, thereby decreasing the probability of misinterpretation.

## Conflict of interest

The authors have stated explicitly that there are no conflicts of interest in connection with this letter.
